# Added value of coronal-T1 W sequence to the lumbar MR imaging protocol for low back pain

**DOI:** 10.7705/biomedica.5845

**Published:** 2022-05-01

**Authors:** Ahmet Nedim Kahraman, Ahmet Vural

**Affiliations:** 1 Department of Radiology, University of Health Science Fatih Sultan Mehmet Training and Research Hospital, Istanbul, Turkey Fatih Sultan Mehmet University University of Health Science Fatih Sultan Mehmet Training and Research Hospital Istanbul Turkey

**Keywords:** low back pain, magnetic resonance imaging, dolor de la región lumbar, imagen por resonancia magnética.

## Abstract

**Introduction:**

Magnetic resonance imaging (MRI) is the most appropriate imaging method to investigate low back pain. As low back pain is very common, a large number of MRI scans are performed.

**Objective:**

To evaluate the extraspinal findings and clinical effect of the T1-weighted spin echo (T1 W SE) coronal sequence added to the lumbar MRI protocol for low back pain.

**Materials and methods:**

In 2015, we added a T1-weighted (T1W) coronal sequence to our routine lumbar MRI protocol. We retrospectively evaluated 969 lumbar MRI images for low back pain performed with this protocol. The extraspinal MRI findings obtained from them were then grouped as associated with low back pain (Category 1) and not associated with low back pain (Category 2). We also evaluated whether the recorded incidental extraspinal findings could be detected on conventional sagittal and axial images.

**Results:**

Ninety-six (63%) of the extraspinal findings were associated with low back pain (Category 1) and 56 (37%), Category 2. Seventy-eight percent of the extraspinal findings were detected only on coronal-T1W images and not on conventional images.

**Conclusion:**

Adding coronal-T1W sequence to the routine protocol of lumbar MRI can help to identify extraspinal findings and guide clinical treatment.

## Resumen

**Introducción.:**

La resonancia magnética (RM) es el método de imágenes diagnósticas más apropiado para investigar el dolor lumbar. Dado que este es muy común, son muchas las resonancias magnéticas de este tipo que se hacen.

**Objetivo.:**

Analizar los hallazgos extra espinales que se pueden detectar al agregar la secuencia coronal T1 W al protocolo de imágenes de RM para el dolor lumbar y evaluar su efecto clínico.

**Materiales y métodos.:**

En el 2015 se agregó una secuencia coronal ponderada en T1W al protocolo de resonancia magnética lumbar de rutina de nuestro hospital. Se evaluaron retrospectivamente 969 imágenes solicitadas en casos de dolor lumbar y realizadas con este protocolo. Los hallazgos obtenidos a partir de dichas imágenes se agruparon luego como asociados con el dolor lumbar (categoría 1) y no asociados con el dolor lumbar (categoría 2). Se evaluó, asimismo, si los hallazgos extra espinales registrados podían detectarse en imágenes axiales y sagitales convencionales.

**Resultados.:**

Noventa y seis (63 %) de los hallazgos extra espinales se asociaron con lumbalgia (categoría 1) y 56 (37 %) correspondieron a la categoría 2. El 78 % de los hallazgos extra espinales se detectaron solo en imágenes coronales-T1W y no en las convencionales.

**Conclusión.:**

La secuencia coronal-T1 W agregada al protocolo de rutina de la resonancia magnética lumbar puede ayudar a detectar afectaciones extra espinales y guiar el tratamiento clínico.

Low back pain is described as the pain or muscle stiffness (sometimes accompanied by leg pain), which occurs in the area up to the gluteal region under the costal border due to the compression of the nerve roots coming out of the spinal cord or as a result of the strain of the muscles or ligaments in the spine ^1^^,^^2^. It is a condition experienced at least once in a lifetime by 84% of adults and more than once in a lifetime by 50% of individuals. Low back pain is the second most common cause of job loss among employees ^3^. It is classified as acute (less than 6 weeks), subacute (6 weeks to 3 months), or chronic (longer than 3 months) ^4^ and it is mainly originated in the spine, the sacroiliac joint, the hips, muscles, ligaments, and nerves ^3^^,^^5^.

After clinical evaluation, patients with low back pain are often referred to MRI for evaluating intervertebral disc pathologies or degenerative diseases of the spine, as it is the most appropriate imaging method to investigate low back pain ^6^^,^^7^. According to the American College of Radiology, sequences commonly used in spine MRI are 2D T1-weighted (T1W) sagittal imaging, 2D T2-weighted (T2W), orT2* sagittal imaging, 2DT1W axial imaging, 2DT2W, orT2* axial imaging ^8^. Besides, the Short Tau Inversion Recovery (STIR) sequence can be performed for an increased view of bone and ligament lesions. Optionally, non-routine MRI sequences including diffusion-weighted imaging, MRI spectroscopy, in-phase and extra-phase MRI, and dynamic contrast-enhanced MRI (perfusion imaging) may be obtained ^8^^-^^10^.

Up to 2015, T1 W and T2W sagittal images and T2W axial images were taken as the standard lumbar MRI protocol at our institution for examining patients with acute or chronic low back pain. In 2015, we added a rapid one- minute T1W coronal sequence that encompassed the entire abdomen with anterior and posterior fields of view to our routine lumbar MRI protocol (table 1) aimed at detecting associated extraspinal pathologies with or without low back pain. Here we performed a retrospective analysis of these images to check the value of incorporating an additional sequence into the routine protocol.

**Table 1:** Lumbar MRI sequence parameters

Sequence	TE, ms	TR, ms	Matrix	Voxel size, mm	Thickness, mm	Gap, mm	FOV, mm	Acquisition time, m:s
Sagittal T1W	15	250	320x320	1x1	4	0.5	320x49 FH320	02:04
Sagittal T2W	100	2350	320x320	1x1	4	0.5	320x49 FH320	02:06
Axial T2W	100	6750	288x288	0.7x0.7	4	0.5	200x200 FH320	02:49
Coronal T1W	18	365	288x288	1x1	6	1	530x265 FH320	01:19

Signa Explorer HD 1.5T GE Healthcare

## Materials and methods

This was a retrospective analysis exempt from Institutional Review Board (IRB) evaluation. It was approved by the institutional research ethics committee for human clinical investigations (permit no: 17073117-050.06) in accordance with the Declaration of Helsinki.

In our cohort study, we evaluated 1,384 lumbar MRI examinations performed between January, 2016, and January, 2018, at our institution. We only included patients referred for lumbar MRI due to low back pain. We excluded patients who had lumbar MRI for reasons other than low back pain, those examined during postoperative follow-up, and those with congenital diseases. We also excluded studies with motion artifacts and patients whose examination could not be completed due to claustrophobia. Finally, we included 969 patients out of 1,384 in the study.

### MRI protocol

Lumbar MRIs were performed with a 1.5 T MRI (Signa Explorer; GE Healthcare Medical Systems, USA) using an 8-channel spine-array superficial coil. If no abnormality was detected in sagittal T1 W, sagittal T2W, and sagittal planes, the axial T2W sequence for intervertebral disc gaps was obtained traditionally from the LI to the sacral level. The plane and field of view (FOV) of the coronal-T1W sequence included the entire diaphragm at the top, the pelvic floor at the bottom, and the entire abdomen from the spine to the anterior abdominal wall, and was extended to include coxofemoral joints and sacrum. MRI parameters are summarized in table 1.

### Image analysis

Two radiologists with an average of 10 years of experience in neuroradiology analyzed both standard and coronal-TIW images. A preliminary evaluation was performed on the basis of T1 W and T2W sagittal images, as well as T2W axial images. Imaging findings were then integrated with the coronal-T1W sequence and a final diagnosis was established. The pathological findings were recorded by consensus. During the spine examination, scoliosis, degenerative disc disease, protrusion or herniation, spinal stenosis, spondyloarthrosis, facet hypertrophy, vertebral corpus corner degeneration, and vertebral bone lesions were evaluated as spinal pathologies. Extraspinal MRI findings including sacroiliac and coxofemoral joint degeneration or sacroiliitis, vascular, genitourinary, and gynecological diseases, were classified as follows: Imaging findings that could be associated with low back pain (Category 1); pathological imaging findings not associated with low back pain (Category 2).

As for renal cysts, simple cysts in Bosniak I stage were classified as Category 1. In the Bosniak kidney cyst classification, size is not a direct criterion for the distinction between stages I and II, but in the revised version, cysts larger than 3 cm should be followed ^11^. For this reason, Bosniak II cysts and above, or those larger than 3 cm in size were considered Category 2. Of the findings related to kidney and ureters, 31 (31/42) were considered to be associated with low back pain while 11 with non-large renal angiomyolipoma and simple cysts were evaluated as not related to low back pain. Although uterine fibroids may cause pain, it is appropriate to classify them as Category 2 given the small size of fibroids (less than 5 cm) ^12^. Biliary dilatation is generally associated with abdominal pain, as it shows obstructive pathologies ^13^.

We determined whether the recorded pathologies were detected on conventional (sagittal T1 W and T2W, and axial T2W) images. We recorded only the pathologies detected in coronal-T1W images.

## Results

Sixty-two percent (n=852) of the patients were male and 38% (n=532) were female. The mean age was 47±19.61 (range 19-89). Of the 969 MRI evaluations, 122 (9.9%) showed 152 different extraspinal MRI findings; 78% (120/152) of them were detected only in the coronal plane. Extraspinal imaging findings are summarized in table 2; 63% of extraspinal imaging findings were considered to be associated with low back pain (Category 1), and 37% were unrelated to low back pain (Category 2); 74% of Category 1 findings and 85% of Category 2 were detected only in the coronal plane (table 2).

**Table 2:** Classification of extraspinal MRI findings

Possible association with low back pain (Category 1)	Unrelated to low back pain (Category 2)
Complex renal cyst (n=16)	Liver hemangioma/cyst (n=18)
Osteoarthritis/degenerative changes (n=16)	Sclerotic nonspecific bone lesion (n=10)
Sacroiliitis (n=15)	Simple renal cyst (n=8)
Renal dilatation (n=15)	Uterine fibroids (n=7)
Bone metastasis (n=9)	Free liquid (n=5)
Ovarian mass/cyst (n=9)	Adrenal mass (n=3)
Sacral insufficiency/fracture (n=5)	Renal angiomyolipoma (n=3)
Dilatation in the biliary tract (n=5)	Muscular atrophy (n=2)
Femoroacetabular impingement (n=3)	
Aortic aneurysm (n=2)	
Retroperitoneal fibrosis (n=1)	

**Figure 1:**
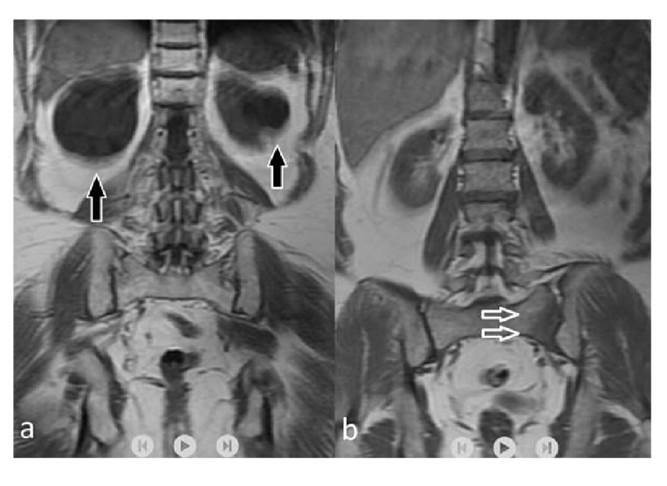
Coronal T1-weighted images show hypointense cysts (arrows) located in both kidneys **(a)**. On the coronal-T 1 weighted sequence **(b)**, a unilateral sacral hypointensity at the level of sacroiliac joints was detected (arrow); this finding is consistent with unilateral sacroiliitis and it was not detectable on conventional MR sequences.

**Figure 2:**
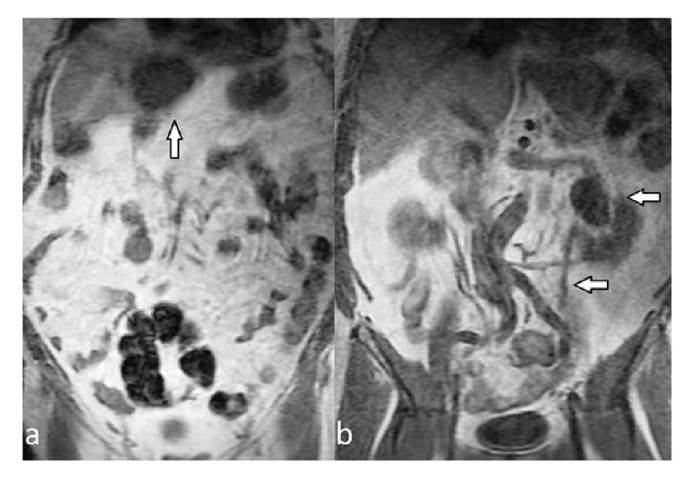
A hypointense mass (arrow) is observed in the liver **(a)**. Coronal T1-weighted images show pelvic dilatation of the left kidney (arrows). There is also dilatation of the left ureter **(b)**.

**Figure 3:**
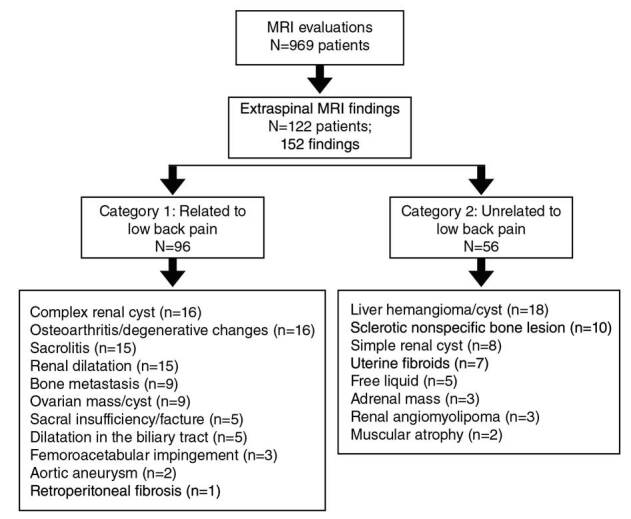
Extraspinal MRI findings summarized in the cohort diagram

All extraspinal abnormalities detected in conventional sagittal T1 W and T2W images appeared in coronal-T1 W images. Most extraspinal MRI findings (27.6%) were related to the kidney or the ureters (42/152); of these, 22 were detectable in the sagittal/axial planes while 20 were detectable only in coronal-T1 W images. Of the pathologies related to the kidney and the ureters, 31 belonged to Category 1 and 11 to Category 2. After the kidney- related pathologies, the most common extraspinal MRI findings in the coronal- T1W sequence were those associated with the pelvic bones including the coxofemoral joints. Osteoarthritis/degenerative changes (n=16), nonspecific bone lesions (n=10), and bone metastases (n=9) were the most common extraspinal MRI findings while 23 were associated with the liver and 20 with the sacroiliac joints. Lesions that may be compatible with cyst or hemangioma in the liver (n=18) and sacroiliitis-related findings (n=15) related to sacroiliac joints were among the common findings (figures 1,2). Also, ovarian cysts/ masses (n=9), uterine fibroids (n=7), and surrenal masses (n=3) were some of the extraspinal MRI findings. Besides, less common MRI findings such as aortic aneurysm (n=2) and retroperitoneal fibrosis (n=1) were also recorded. The classification of extraspinal MRI findings is shown in tables 2 and 3. The grouping and number of patients in each group is also summarized in the cohort diagram (table 3 and figure 3).

**Table 3:** Extraspinal MRI findings by region

Pathology region	Number of patients	Seen in axial/sagital plan	Only seen in coronal-T1W	Category 1	Category 2
Kidney and ureter	42	22	20	31	11
Pelvic bones	38	4	34	28	10
Liver	23	0	23	0	23
Sacroiliac joint	20	3	17	20	0
Internal genitals	16	0	16	9	7
Adrenal	3	0	3	0	3
Aorta	2	1	1	2	0
Other	8	2	6	6	2
Total	152	32	120	96	56

Of the 42 extraspinal findings in kidneys and ureters, 22 appeared in the axial and sagittal planes, 20 only in the coronal T1W sequence while 39 of 42 pathologies could be seen in the coronal T1W sequence. The coronal T1W sequence was more successful (p=0.01, i.e., statistically significantly) according to the evaluation with the Mcnemar test for the detection of kidney and ureter extraspinal findings. There were 38 subjects with extraspinal findings in the pelvic bones. In 34 subjects, the findings were visible only on coronal T1W images while only four subjects had pelvic bone pathology in axial and sagittal conventional images, and 37/38 (97%) of the pelvic bone pathologies could be detected in coronal T1W images. Coronal T1W sequence was found to be more successful in detecting pelvic bone pathologies (p=0.01, i.e., statistically significantly). Similarly, coronal T1W images were found to be more successful in the detection of extraspinal findings in the liver, sacroiliac joints, and internal genital organs (p<0.05, i.e., statistically significant).

## Discussion

Based on our results, the use of the coronal-T1 W sequence allowed for the detection of MRI findings not visible in the sagittal or axial planes in 4.5% of patients (44/969) significantly altering their diagnostic workflow and prognosis. Additionally to conventional lumbar MRI performed for low back pain, it was possible to get coronal-T1W images including the site from the spine to the abdominal anterior wall level, as well as findings in the musculoskeletal system and the abdominopelvic region. Thus, the major extraspinal causes of low back pain can be identified as problems affecting the sacroiliac joints, coxofemoral joints, or pelvic bones. Most of these problems cannot be detected using the conventional lumbar MRI examination protocol. In many clinics, a coronal STIR sequence has been added to the lumbar MRI protocol to detect sacroiliitis, which is one of the major causes of low back pain ^14^. The use of a coronal-T1W sequence instead of a coronal STIR sequence appears to be highly successful in the diagnosis of sacroiliitis. Instead of using a coronal STIR sequence for imaging only the spine and sacroiliac joints, many pathologies associated with low back pain and sacroiliitis can be visualized using a coronal-T1W sequence involving the entire abdomen. Also, many pathologies not associated with low back pain can significantly alter the diagnostic process and prognosis of the patient.

Of the 152 extraspinal MRI findings detected in our study, 33 (22%) were detectable on conventional images, while 129 (78%) were visible only on coronal-T1W images, and 42 of the pathological extraspinal imaging findings were related to the kidneys and ureters. It is possible to detect many renal imaging findings in the axial plane, but the coronal plane allows for easier detection and characterization. The collector system enlargement from renal pathologies, complex cysts (Bosniak II and above), and cysts larger than 3 cm were accepted as associated with low back pain ^15^. After renal diseases, liver lesions, unilateral or bilateral sacroiliitis, coxofemoral disease, and iliac bone lesions were the most common pathological extraspinal MRI findings in our study. Findings compatible with metastasis from bone-derived pathologies, sacroiliitis, coxofemoral pathologies, and biliary dilatation were classified as possible pathologies associated with low back pain.

Gleeson *et al*. concluded that in a study evaluating sacroiliac joints and sacrum with coronal-STIR sequence added to lumbar MRI for low back pain, in a few cases (2%) diagnostic evaluation improved ^16^. More recently, Gupta *et al*. investigated the value of adding the coronal-STIR sequence to lumbar MR imaging in a smaller patient population. They evaluated extraspinal findings (inflammatory sacroiliitis, sacroiliac joint degeneration, sacral stress fracture, muscle injury) retrospectively. They reported that coronal-STIR imaging may provide an additional diagnosis in 6.8% of patients and should be included in the routine lumbar spine MRI protocol ^17^. Similarly, other studies evaluating the diagnostic efficacy of the coronal-STIR sequence added to the conventional lumbar MR imaging have reported extraspinal incidental findings including vascular, genitourinary, gastrointestinal, musculoskeletal, and oncological pathologies ^18^^-^^23^. In these studies, the coronal-STIR sequence was performed for the backbone region. In our study, we added coronal-T1W imaging and performed a sequence planning that would cover not only the spinal region but also the entire abdominal region up to the anterior abdominal wall. Adding the coronal-STIR sequence to lumbar MRI in patients with low back pain is becoming more common. The most important reason for this is that sacroiliitis, which is the most common cause of unresolved low back pain, can be revealed. In our study, we found that coronal-T1W imaging could successfully detect sacroiliitis, as well as more extraspinal findings associated with low back pain or not. We also wanted to demonstrate the advantage of T1W imaging for better anatomy screening. The results are more prominent than in general screening, especially in sclerotic bone lesions ^24^. In sequence planning, we have tried to optimize a sequence that lasts shorter than the coronal-STIR sequence for the spine alone but allows us to evaluate the whole abdomen in the foreground. One of the disadvantages of the coronal-STIR sequence is its inability to evaluate frequently observed signal changes in the vertebra corpus corners. T1W images provide more valuable information in the evaluation of trauma, degeneration, fatty bone marrow, and sclerotic signal changes. Similarly, T1W images are more beneficial than the STIR sequence in sacroiliitis with the dominant sclerotic component ^25^^,^^26^.

We observed that some of the extraspinal MRI findings had significantly led to an earlier diagnosis algorithm, which is important for the prognosis and treatment plan. On the other hand, the majority of the findings were benign pathologies and did not affect the prognosis. In this sense, over-diagnosis may cause unnecessary anxiety and lead to additional exams. The most important limiting factor in our study was the quality of abdominal images obtained with surface coils optimized for lumbar MR imaging. However, our aim was not the detection of all abdominal pathologies with high sensitivity. On the other hand, we wanted to detect as many gross extraspinal lesions as possible by adding a minute-long T1W sequence to the spinal imaging. Also, we could have overlooked small lesions due to the high cross-sectional thickness and spacing used to optimize the sequence time and the lack of direct comparison with the coronal-STIR sequence. We believe that a study of both the coronal- STIR sequence and coronal-T1W images would provide valuable information.

In conclusion, we believe that adding coronal-T1W images to the routine lumbar MRI for patients presenting with low back pain may help detect extraspinal MRI findings associated with low back pain and reveal additional findings that improve the prognosis of the patient without adding cost. Since the same coil is used and the procedure takes only an additional minute, it provides benefits by detecting incidental lesions in the abdomen with no extra burden for the patients.
